# Predicting weight loss success on a new Nordic diet: an untargeted multi-platform metabolomics and machine learning approach

**DOI:** 10.3389/fnut.2023.1191944

**Published:** 2023-08-01

**Authors:** Kristina Pigsborg, Valdemar Stentoft-Larsen, Samuel Demharter, Mona Adnan Aldubayan, Alessia Trimigno, Bekzod Khakimov, Søren Balling Engelsen, Arne Astrup, Mads Fiil Hjorth, Lars Ove Dragsted, Faidon Magkos

**Affiliations:** ^1^Department of Nutrition, Exercise and Sports, University of Copenhagen, Frederiksberg, Denmark; ^2^Abzu ApS, Copenhagen, Denmark; ^3^King Saud bin Abdulaziz University for Health Sciences, College of Applied Medical Sciences, Riyadh, Saudi Arabia; ^4^Department of Food Science, University of Copenhagen, Frederiksberg, Denmark; ^5^Obesity and Nutritional Sciences, Novo Nordisk Foundation, Hellerup, Denmark

**Keywords:** precision nutrition, metabolomics, obesity, new Nordic diet, machine learning

## Abstract

**Background and aim:**

Results from randomized controlled trials indicate that no single diet performs better than other for all people living with obesity. Regardless of the diet plan, there is always large inter-individual variability in weight changes, with some individuals losing weight and some not losing or even gaining weight. This raises the possibility that, for different individuals, the optimal diet for successful weight loss may differ. The current study utilized machine learning to build a predictive model for successful weight loss in subjects with overweight or obesity on a New Nordic Diet (NND).

**Methods:**

Ninety-one subjects consumed an NND *ad libitum* for 26 weeks. Based on their weight loss, individuals were classified as responders (weight loss ≥5%, *n* = 46) or non-responders (weight loss <2%, *n* = 24). We used clinical baseline data combined with baseline urine and plasma untargeted metabolomics data from two different analytical platforms, resulting in a data set including 2,766 features, and employed symbolic regression (QLattice) to develop a predictive model for weight loss success.

**Results:**

There were no differences in clinical parameters at baseline between responders and non-responders, except age (47 ± 13 vs. 39 ± 11 years, respectively, *p* = 0.009). The final predictive model for weight loss contained adipic acid and argininic acid from urine (both metabolites were found at lower levels in responders) and generalized from the training (AUC 0.88) to the test set (AUC 0.81). Responders were also able to maintain a weight loss of 4.3% in a 12 month follow-up period.

**Conclusion:**

We identified a model containing two metabolites that were able to predict the likelihood of achieving a clinically significant weight loss on an *ad libitum* NND. This work demonstrates that models based on an untargeted multi-platform metabolomics approach can be used to optimize precision dietary treatment for obesity.

## Introduction

Obesity has reached pandemic proportions over the last decades and is a major risk factor for several co-morbidities including cardiovascular diseases, dyslipidemia, hypertension, insulin resistance, type 2 diabetes, non-alcoholic fatty liver, and cancer (1–5). Scientists have long searched for the optimal diet to treat obesity, and the view on which diet is best has shifted over time (6). The 1980s and 1990s have seen a focus on low-fat diets whereas recently, the focus has been placed on limiting sugar consumption and carbohydrates in general, but also on adopting a more plant-based, fiber-rich diet.

Different diets have variable efficacy in reducing body weight in the short term, but none of them is superior to others in the long term; in fact, no diet can provide an average efficacy above 10% (7–10). However, there is a large inter-individual variation in response to the same dietary treatment, with different individuals experiencing different rates of weight loss and eventually some achieving large amounts of weight loss and others having none or even gaining weight (11–13). It is, however, possible, that different groups of individuals will succeed on different diets, emphasizing the need for precision nutrition (14). The reasons for this inter-individual variability in weight loss responses are not well known but likely have a metabolic nature (15). Differences in metabolic processes might be reflected in the metabolome, as metabolites are the end-products of cellular regulatory processes and their levels in different biological matrices reflect the biological response to genetic, microbial, or environmental changes (16–18).

Metabolomics – like all other ‘omics’ techniques – produces extensive datasets and a wealth of information, but at the same time presents the challenge of high dimensionality of datasets, where the number of variables far exceeds the number of subjects. Moreover, no analytical platform is able to capture the whole metabolome wherefore a multi-platform approach is increasingly applied to larger intervention studies. Moving from large and complex datasets to a better understanding of metabolic responses to facilitate future application in clinical settings requires sophisticated data analytics tools such as machine learning techniques. Nevertheless, most machine learning algorithms produce black-box models that can be difficult to understand and interpret. Symbolic regression is particularly suitable for scenarios where the number of features in the model should be kept at a minimum when their interpretation and interactions are of primary interest; this is exemplified by the QLattice algorithm, which has shown promising results with small datasets (19, 20) and in the context of omics-based biomarker identification (21). This is, to our knowledge, the first time QLattice has been utilized in human nutrition research.

In the present study, we acquired and analyzed metabolomics datasets at baseline from the Shop Model for Optimal Dietary Adherence (SHOPUS) study (22) and used QLattice on a combination of several metabolomics datasets and clinical study data to predict weight loss success for subjects with overweight or obesity following a New Nordic diet (NND).

## Methods

### Study design and participants

The SHOPUS study was a 26 week unblinded, parallel, randomized, controlled dietary intervention trial (ClinicalTrials.gov number NCT01195610). The study has been reported in detail previously (22). Briefly, subjects with increased waist circumference (>94 cm for men and >80 cm for women) were randomized in a 3:2 ratio to either the NND or the control diet (Average Danish Diet, ADD), respectively. In total, 181 participants were assigned to the two diet groups and after 26 weeks, 91 and 56 participants completed the study in the NND and ADD arms, respectively (see Supplementary Figure S1). Participants were encouraged to maintain their regular physical activity habits throughout the intervention period.

### Ethics statement

The ethics committee of the Capital Region of Denmark approved the trial (H-3-2010-058) and written informed consent was obtained from each subject before participation. The study was carried out in accordance with the principles of the Declaration of Helsinki and was pre-registered at clinicaltrials.gov (NCT01195610).

### Responders and non-responders

**Figure 1:**
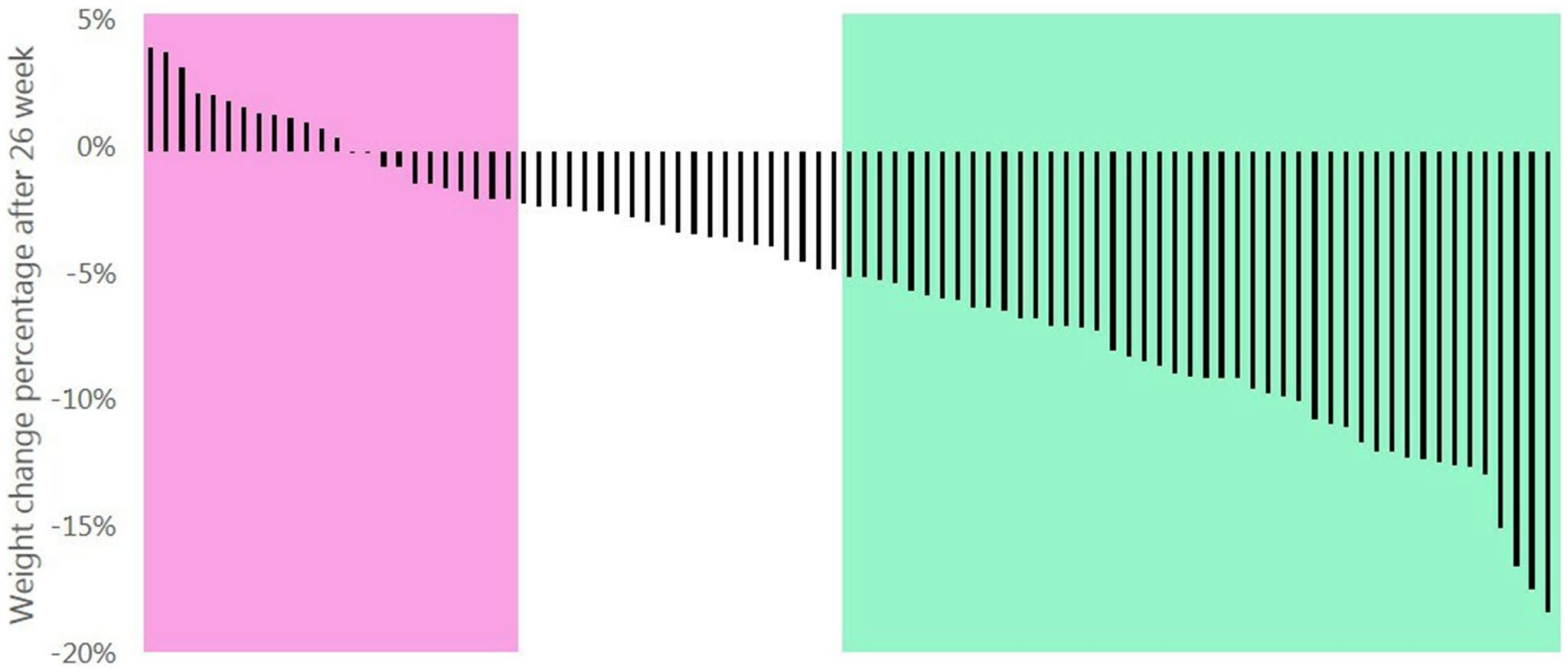
Percentage weight change of the participants completing the 26 weeks intervention following a New Nordic diet. Responders had a weight loss ≥5% (green area) and non-responders had a weight loss <2% (pink area).

Subjects who completed the study were classified as “responders” if they had lost ≥5% of their initial body weight or “non-responders” if they lost <2% of their initial body weight (23). Subjects who lost between 2%–5% of their initial body weight were not included in the primary analysis (see Figure 1).

### Intervention diet

Subjects followed guidelines for eating in accordance with NND principles and collected (free of charge) all their groceries in the shop developed specifically for the study within the premises of the Department of Nutrition, Exercise and Sports at the University of Copenhagen. Over the 26 weeks, the diets were consumed *ad libitum* but the intention was for participants to lose weight. The NND (24) was a whole-food dietary paradigm characterized by higher contents of dietary fiber, whole grain, fruit, nuts, and vegetables, whereas the ADD was designed to match the macronutrient composition of an average Danish diet (22). All participants underwent a 1 week run-in period on the ADD to get familiar with the supermarket shop. Throughout the subsequent 26 weeks, participants had regular consultation meetings with a dietician for guidance on diet and behavior, in addition to cooking classes and workshops. Dietary intake was assessed using 3 day weighed food records at week 0 and week 26 where participants reported all consumed foods and beverages throughout three consecutive days including one weekend day. Compliance with the diet was evaluated by the study dieticians on a scale from 1 = “very bad” to 5 = “very good” during the visit days, based on the extent to which participants integrated the dietary advice into their everyday diet. On the same scale and time points, the participants rated their satisfaction with the diet.

### Clinical outcomes

Height was measured at screening and fasting body weight was measured at baseline, week 12 and week 26. Waist and hip circumferences, resting blood pressure, and body composition (fat mass and lean mass, by using dual-energy x-ray absorptiometry) were also measured at the three time points. Fasting blood samples were drawn at baseline and week 26 and analyzed for triglyceride, total cholesterol, high-density lipoprotein (HDL)-cholesterol, low-density lipoprotein (LDL)-cholesterol, glucose, and insulin. Insulin resistance was evaluated by using the homeostasis model assessment (HOMA-IR) score, calculated as follows: fasting plasma glucose (mmol/L) × fasting plasma insulin (mU/mL)/22.5 (25). Furthermore, a 2 h oral glucose tolerance test (OGTT, 75 g glucose diluted in 250 mL water) was conducted at baseline and week 26, from which the Matsuda index of insulin sensitivity was calculated as follows: 10,000/square root of {[fasting glucose (mg/dL) × fasting insulin (mU/mL)] × [mean glucose (mg/dL) × mean insulin (mU/mL)]}, where “mean” refers to the average concentrations during the OGTT (26). Participants also performed a 24 h urine collection before each of the three visits. After the intervention period (week 26), they had follow-up visits with a dietician at weeks 52 and 78 where body weight was measured again (in a non-fasted state). During the 52 week follow-up, all subjects were encouraged to continue following the NND and to exercise more, but with no reinforcement (27).

### LC–MS untargeted metabolomics profiling and data preprocessing

Urine and plasma samples were profiled with untargeted metabolomics approaches on an ultra-performance liquid chromatography (UPLC) system coupled to quadrupole time-of-flight (Premier QTOF) mass spectrometer (MS) (Waters Corporation, Manchester, United Kingdom) in both positive and negative ionization modes, as previously described for urine (28) and plasma (29) samples at the Department of Nutrition, Exercise and Sports (University of Copenhagen).

The different LC–MS datasets were preprocessed individually in R (ver. 4.2.1) using the package XCMS (30) with the parameters listed in Supplementary Table S1 for both plasma and urine data in both positive and negative ionization modes. Here, a list of features was produced and defined in a three-dimensional list containing retention time (RT), mass-to-charge ratio (m/z), and the measured signal intensity (peak height). Lists of different features were obtained after preprocessing and irrelevant features in each dataset were removed before statistical data analysis by the following criteria: (1) features present in blank samples, (2) features eluting before 0.3 min or after 6.5 min, (3) features present in <30% of samples in each subgroup, (4) potential duplicate features or isotopes annotated by the CAMERA package (31), and (5) features showing a coefficient of variance >0.7 in the quality control samples. Intra-batch correction within each plate was performed to reduce the effect of analytical drift and no inter-batch correction was performed, as this was deemed unnecessary (see Supplementary Figures S2A–D).

Metabolites measured by LC–MS were putatively annotated using their m/z, RT, and mode, and then annotated at level 1 as described by the Metabolomics Standard Initiative (32) when spectra were identical for authentic standards and the metabolites (Supplementary Figures S3, S4), recorded by UHPLC (Waters Acquity)-coupled tandem mass spectrometry (Vion IMS QTOF mass spectrometer, Waters Corporation, Manchester, United Kingdom) at ionization energies of 10, 20 and 30 eV, as described in Supplementary Table S2.

### NMR metabolomics analysis and preprocessing

One-dimensional (1D) proton nuclear magnetic resonance (1H NMR) spectroscopic analysis of urine and plasma samples was performed at the Department of Food Science (University of Copenhagen) using a Bruker Avance III 600 spectrometer (Bruker Biospin Gmbh, Rheinstetten, Germany) operating at a Larmor frequency of 600.13 MHz for protons, equipped with a double tuned cryo-probe (TCI) set for 5 mm sample tubes and a cooled autosampler (SampleJet). Proton NMR spectra were acquired on all plasma samples using the Carr-Purcell Meiboom-Gill (CPMG) experiment (which provides semi-quantitative data) (33) and urine samples were measured using the NOESY-presat pulse sequences from Bruker’s library (possible urine dilution differences were taken into account by normalizing to unit length for urine) (34). The plasma experiments were performed at 310 K and the urine experiments at 300 K. The automation program controlling sample measurements included the acquisition routines for locking, automatic tuning and matching, shimming, pulse calibration, and optimized pre-saturation power for each sample, as well as automatic data processing including Fourier transformation (FT) of FID, with a Lorentzian line-broadening of 0.3 Hz before FT, phasing, and baseline correction (Topspin ver. 2.1 and 3.5 PL6; Bruker Biospin Gmbh, Rheinstetten, Germany). Raw NMR spectra were converted to a metabolite concentration table using SigMa software (35). The processing included reference alignment towards the TSP signal at 0.0 ppm, pre-alignment of larger spectral regions using the *icoshift* method (36) followed by interval recognition. Spectra were divided into smaller regions of Signature Signals (SS) of known human blood metabolites, Signals of Unknown Spin systems (SUS), and BINS representing complex regions containing unresolved signals of more than one metabolite. After interval recognition, SigMa quantified SS and SUS variables using a one-component Multivariate Curve Resolution (MCR) model with non-negativity constraints (37, 38), and BINS were quantified using integration by summation.

### Statistical analysis

Statistical comparisons between responders and non-responders were performed by a two-sample, unpaired *t*-test if data were normally distributed or by a Mann–Whitney test if data were not normally distributed. Health outcomes and metabolites from the model were tested for correlation by using Spearman coefficients. A value of *p* <0.05 was considered significant. The statistical analysis was performed in R (ver. 4.2.1).

### Data integration and machine learning strategies

**Figure 2:**
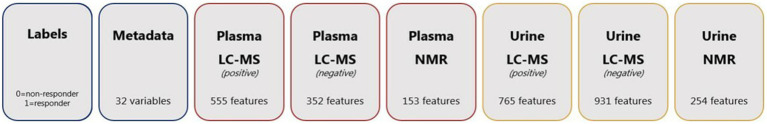
Data sets used in this study: label information corresponding to each subject (0 = non-responder and 1 = responder), metadata including clinical variables, metabolomics measurements of LC–MS (both positive and negative ionization mode) and NMR analysis.

The final dataset for the predictive model was composed of 7 data sets: urine and plasma samples analyzed with LC–MS in both positive and negative mode, urine and plasma samples analyzed with NMR, and metadata (Figure 2). A total of 2,766 features were included in the final data set. Subjects with >5 missing values of the features were removed from the dataset; for subjects with ≤5 missing values, the missing values were imputed with a mean value of the feature’s intensity for the remaining subjects.

Before training the model, the dataset was split so that 60% was used as a training set and the remaining 40% was left out and used as a test set. Furthermore, the training and test datasets were stratified to conserve the ratio of responders to non-responders as the responders represented about two-thirds of the total sample. Predictive modeling was constructed by QLattice in Python (ver. 3.0.4) using the Feyn package (39). QLattice is a novel machine learning method based on symbolic regression (40). Symbolic regression is a subfield of machine learning that seeks to discover mathematical expressions that represent a relationship between input variable *X* and a target variable *Y* without any prior information on the functional form of the relationship (21).

The model was set up to solve the classification problem of separating responders from non-responders (responder = 1, non-responder = 0) using the Bayesian information criterion (BIC) to ensure that the resulting models generalize well from the training set to the test set (21). Due to the low number of subjects relative to the high number of variables and the associated risk of false discovery, complexity restrictions were put on the size of the mathematical expressions. Specifically, the maximal complexity configuration in the QLattice was tested with settings of 3, 4, or 5 corresponding to the combined number of inputs and mathematical operators allowed (such as add, multiply, log, etc.). The different datasets were tested individually and also as a merged dataset. Furthermore, receiver-operating characteristic (ROC) curve analysis was performed to assess the discriminative accuracy of the models. The area under the curve (AUC) is a quantitative measure of the predictive ability and varies from 0.5 for a random prediction to 1.0 for a perfect prediction.

## Results

### Phenotyping and characteristics of responders and non-responders and effects of NND

Among subjects randomized to NND, 46 were categorized as responders with a body weight loss between 5.0 and 18.8% of their initial weight, and 24 were categorized as non-responders with a weight change between −1.9% (weight loss) to +4.1% (weight gain) (see Figure 1). At baseline, the two groups were comparable in anthropometric measures, glycemic control, and lipid profile, but differed in age with responders being ~8 years older than non-responders (Table 1).

**Table 1:** Baseline characteristics of the responders (≥5% weight loss) and non-responders (<2% weight loss) following a new Nordic diet for 26 weeks.

	Responders (*n* = 46)	Non-responders (*n* = 24)	*p*-values
Sex, %females	71.7	66.7	0.67
Age, *y*	47.3 ± 13.0	39.4 ± 11.0	0.01
Anthropometry			
Body weight, kg	89.9 ± 16.3	91.5 ± 18.0	0.72
Body mass index, kg/m^2^	30.6 ± 4.6	30.0 ± 4.4	0.59
Waist circumference, cm	100.0 ± 12.2	100.7 ± 12.8	0.84
Hip circumference, cm	111.2 ± 9.7	109.8 ± 11.2	0.78
Fat mass, kg	37.2 ± 11.1	37.3 ± 11.4	0.99
Body fat, %	41.2 ± 8.0	40.2 ± 6.3	0.55
Systolic blood pressure, mmHg	125 ± 14	122 ± 14	0.45
Diastolic blood pressure, mmHg	82 ± 9	82 ± 9	0.60
Glycaemic markers			
Fasting glucose, mmol/L	5.3 ± 0.5	5.2 ± 0.4	0.27
Fasting insulin, pmol/L	60 (42-83)^a^	72 (40–103)	0.51
HOMA-IR	2.5 ± 1.7	3.0 ± 2.5	0.31
2 h glucose, mmol/L	5.8 ± 1.5	5.9 ± 1.8	0.71
Matsuda index	5.4 ± 2.7	5.4 ± 3.4	0.97
Lipid profile markers			
Triglycerides, mmol/L	1.0 (0.7–1.4)^a^	1.0 (0.7–1.2)	0.88
Total cholesterol, mmol/L	4.9 ± 0.9^a^	4.5 ± 0.9	0.09
HDL cholesterol, mmol/L	1.2 ± 0.3^a^	1.2 ± 0.3	0.92
LDL cholesterol, mmol/L	3.2 ± 0.9^a^	2.8 ± 0.7	0.07
Urine nitrogen, g/day	14.5 ± 4.0	15.5 ± 3.8	0.35

Data are presented as means ± SD or median (IQR). BMI, body mass index; HDL, high-density lipoprotein; LDL, low-density lipoprotein.

a *n* = 45. *p*-values are shown only for descriptive purposes and not for hypothesis testing. The homeostatic model assessment of insulin resistance (HOMA-IR) was calculated as fasting plasma glucose (mmol/L) × fasting plasma insulin (mU/mL)/22.5. The matsuda index was calculated as 10,000/sqrt{[fasting glucose (mg/dL) × fasting insulin (mU/mL)] × [mean glucose (mg/dL) × mean insulin (mU/mL) concentrations during oral-glucose-tolerance test]}.

The responders lost 8.3 ± 3.3 kg during the intervention, of which 7.5 ± 2.7 kg was fat mass and 0.5 ± 1.3 kg was lean mass, while the non-responders gained 0.5 ± 1.8 kg (they lost 0.3 ± 1.7 kg fat mass and gained 1.0 ± 1.2 kg lean mass). Most other health outcomes related to weight loss were not different at baseline between responders and non-responders but differed after the intervention (Table 2). Accordingly, fasting glucose, insulin, and total and LDL-cholesterol concentrations decreased more in responders than in non-responders. Furthermore, urine nitrogen levels increased slightly for both groups but mostly for the responders (*p* = 0.09 for the change) but this did not result in significant differences between groups after the 26 week intervention.

**Table 2:** Changes in weight loss-related health outcomes over the 26 week intervention.

	Responders (*n* = 46)	Non-responders (*n* = 24)	*p*-values
Anthropometry			
Body weight, kg	−8.3 ± 3.3***	0.5 ± 1.8	<0.00001
BMI, kg/m^2^	−3.0 ± 1.6***	0.1 ± 0.5	<0.00001
Waist circumference, cm	−7.5 ± 3.9***	0.4 ± 4.1	<0.00001
Hip circumference, cm	−5.3 ± 5.1***	−0.3 ± 5.1	0.0005
Fat mass, kg	−7.5 ± 2.7***	−0.3 ± 1.7	<0.00001
Lean mass, kg	−0.5 ± 1.3**	1.0 ± 1.2***	<0.001
Body fat, %	−5.2 ± 2.4***	−0.5 ± 1.4	<0.00001
Systolic blood pressure, mmHg	−7 ± 8***	−4 ± 9	0.16
Diastolic blood pressure, mmHg	−5 ± 7***	1 ± 7	0.001
Glycaemic markers			
Fasting glucose, mmol/L	−0.3 ± 0.3***	−0.1 ± 0.4	0.03
Fasting insulin, pmol/L	−23 ± 38***^a^	0 ± 21	0.009
HOMA-IR	−0.9 ± 0.8***	−0.1 ± 1.6	0.03
2 h glucose, mmol/L	−0.0 ± 1.3	0.1 ± 1.5	0.73
Matsuda index	1.6 ± 2.1***	−1.0 ± 2.4	<0.00001
Lipid profile			
Triglycerides, mmol/L	−0.1 ± 0.2**^a^	0.0 ± 0.3	0.12
Total cholesterol, mmol/L	−0.4 ± 0.7**^a^	0.1 ± 0.4	<0.001
HDL cholesterol, mmol/L	0.0 ± 0.2^a^	0.0 ± 0.2	0.99
LDL cholesterol, mmol/L	−0.4 ± 0.6**^a^	0.1 ± 0.4	<0.001
Urine nitrogen, g/day	1.8 ± 6.3	1.2 ± 4.6	0.64

Data are presented as means ± standard deviation or median (IQR). BMI, body mass index; HDL, high-density lipoprotein; LDL, low-density lipoprotein.

a *n* = 45, *p < 0.05, **p < 0.005, and ***p < 0.0005 changes from baseline. The homeostatic model assessment of insulin resistance (HOMA-IR) was calculated as fasting plasma glucose (mmol/L) × fasting plasma insulin (mU/mL)/22.5. The matsuda index was calculated as 10,000/sqrt{[fasting glucose (mg/dL) × fasting insulin (mU/mL)] × [mean glucose (mg/dL) × mean insulin (mU/mL) concentrations during oral-glucose-tolerance test]}.

### Predicting the success of weight loss on the new Nordic diet

**Figure 3:**
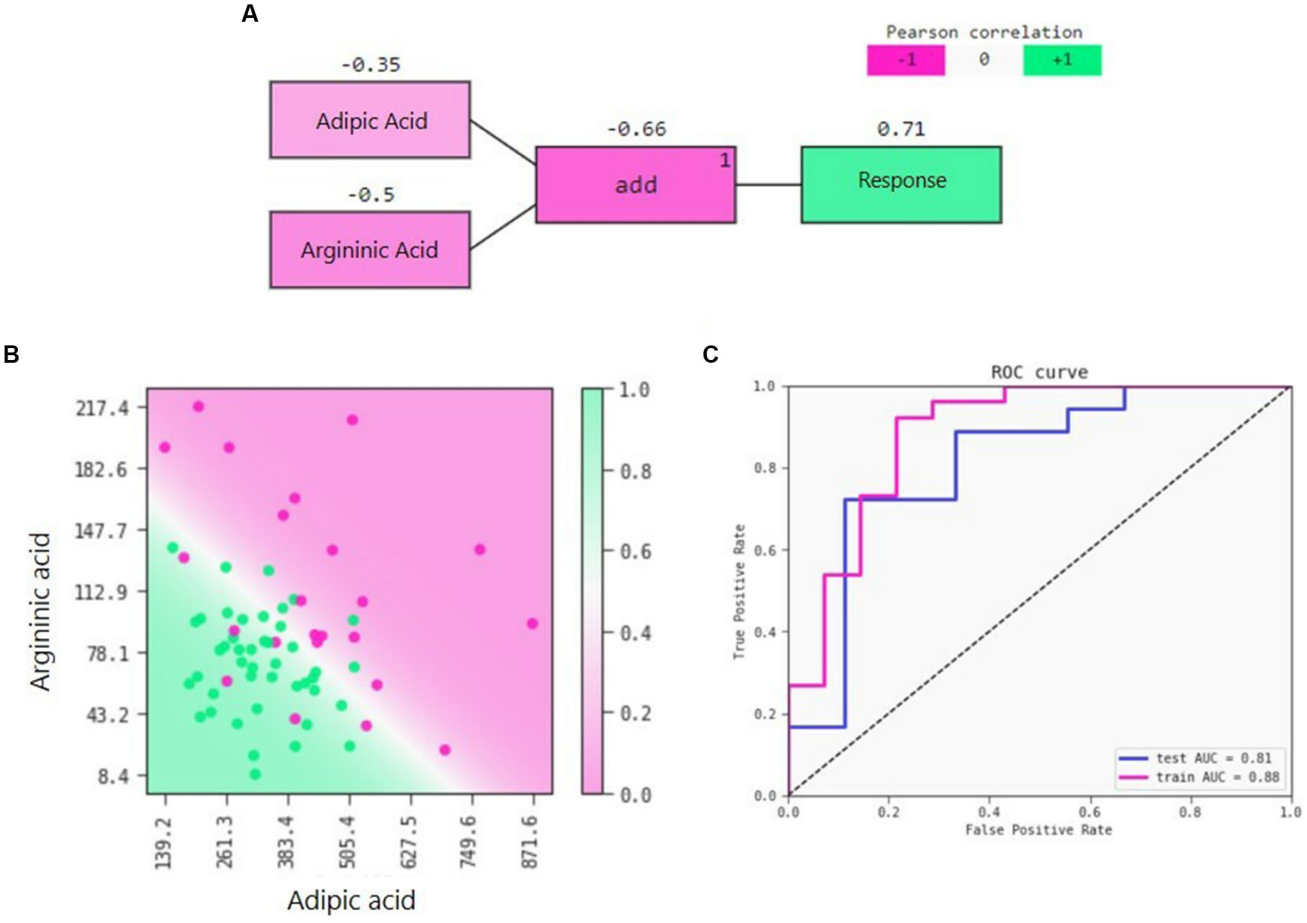
**(A)** Model signal path for the success of weight loss on an NND diet, **(B)** 2D response of the model predictions with training and test data overlaid. The decision boundary separates the response areas. The dots represent the subjects of the classified responders (green) and non-responders (pink) whereas the background represents the model’s prediction (1 = responder, 0 = non-responder), and **(C)** Receiver operator characteristic (ROC) for training and test set.

QLattice was employed to develop a predictive model for weight loss solely based on baseline variables (i.e., prior to the intervention). The symbolic regression approach identified adipic acid and argininic acid two metabolites (both measured in urine by LC–MS) as the components of the model yielding the best discrimination between responders and non-responders as measured by the AUC. The best model is a logistic regression with an additional interaction of the two metabolites (Figure 3A). The model shows that lower levels of both adipic acid and argininic acid increase the likelihood of having successful weight loss on the NND (Figure 3B). The function of the model can be written as:

$$\mathrm{Response}=\frac{e^{0.013.\mathrm{adipic acid}-0.048.\mathrm{argininic acid}+9.4}}{1+e^{0.013.\mathrm{adipic acid}-0.048.\mathrm{argininic acid}+9.4}}$$

The model performed robustly from the training dataset with a ROC-AUC of 0.88 to the test dataset with a ROC-AUC of 0.81 (Figure 3C) and a precision of 0.89 and 0.82, respectively. Each of the metabolites individually did not predict the response better than they did together (adipic acid AUC 0.69 and argininic acid AUC 0.77). In a sensitivity analysis, when testing the performance of the model after 12 weeks (as opposed to 26) and with a cut-off of 2% weight loss for responders (as opposed to 5%), we obtained a ROC-AUC of 0.71 and a precision of 0.84. Finally, other machine-learning tools such as Random Forest, Extreme Gradient Boosting, *k*-means cluster, and partial least squares-discriminant analysis were also tested, but did not perform better than the QLattice.

### Associations at baseline and changes in response to NND

**Figure 4:**
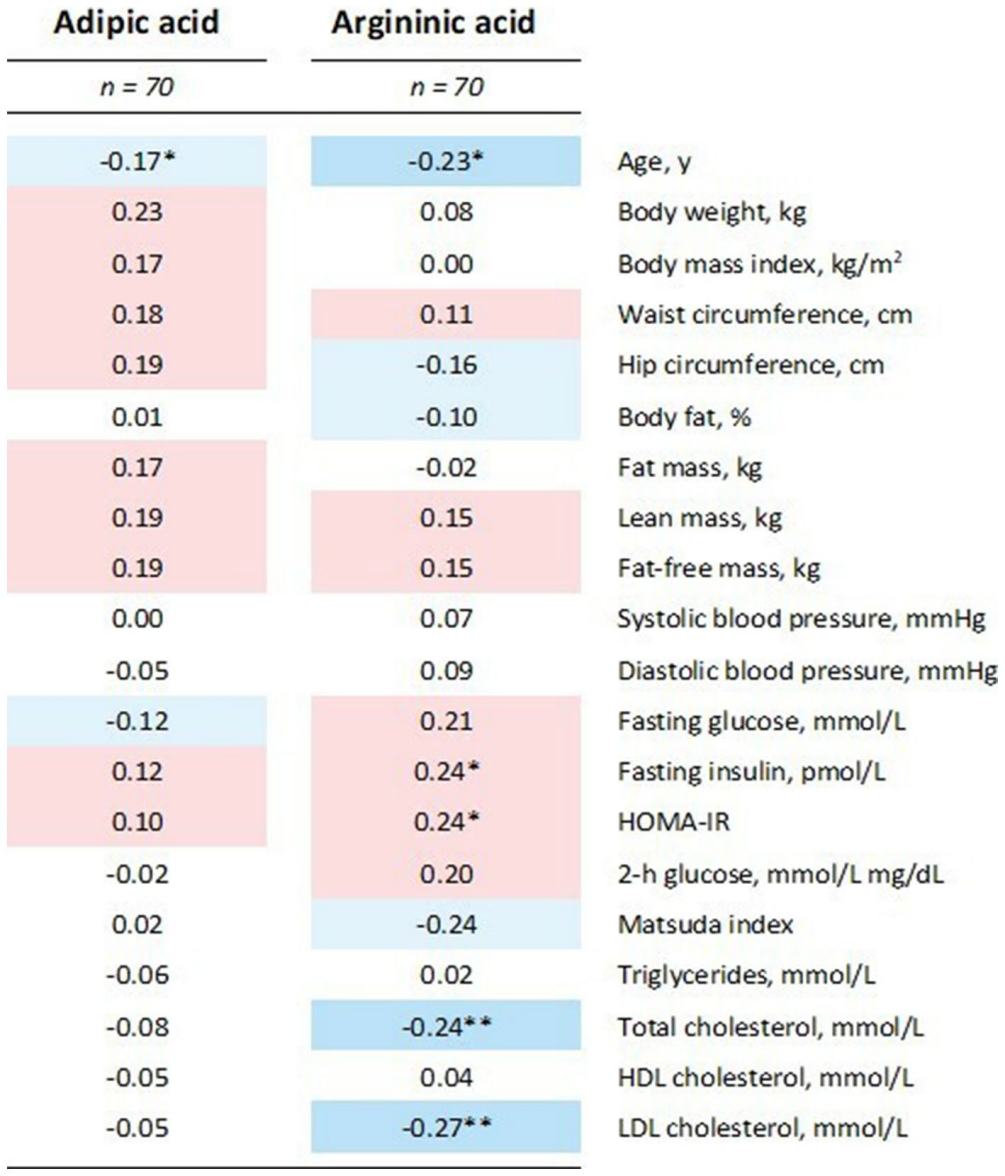
Heatmap of adipic acid and argininic acid correlation with baseline levels of clinical variables using Pearson correlations. Intensity of the blue and red colors indicate the strength of negative and positive correlations, respectively. *p < 0.05 and **p < 0.005. HOMA, homeostatic model assessment for insulin resistance; HDL, high-density lipoprotein; LDL, low-density lipoprotein.

As expected, adipic acid and argininic acid were both negatively correlated to the weight loss response (i.e., successful or not; *r* = −0.35, *p* = 0.03, and *r* = −0.49, *p* = 0.001, respectively). The two metabolites did not show any strong correlation to any of the clinical outcomes at baseline (Figure 4). A list of the 10 most correlated features for both metabolites is shown in Supplementary Tables S3, S4. Here, α-keto-δ-guanidinovaleric acid showed the strongest correlation to argininic acid (*r* = 0.71).

When weight change after 26 weeks for the responders and non-responders was evaluated as a continuous variable, there was no relationship with adipic acid (*r* = −0.15, *p* = 0.22) but argininic acid was inversely correlated to weight change (*r* = −0.40, *p* < 0.001), also after adjusting for age (*r* = −0.35, *p* = 0.004).

**Figure 5:**
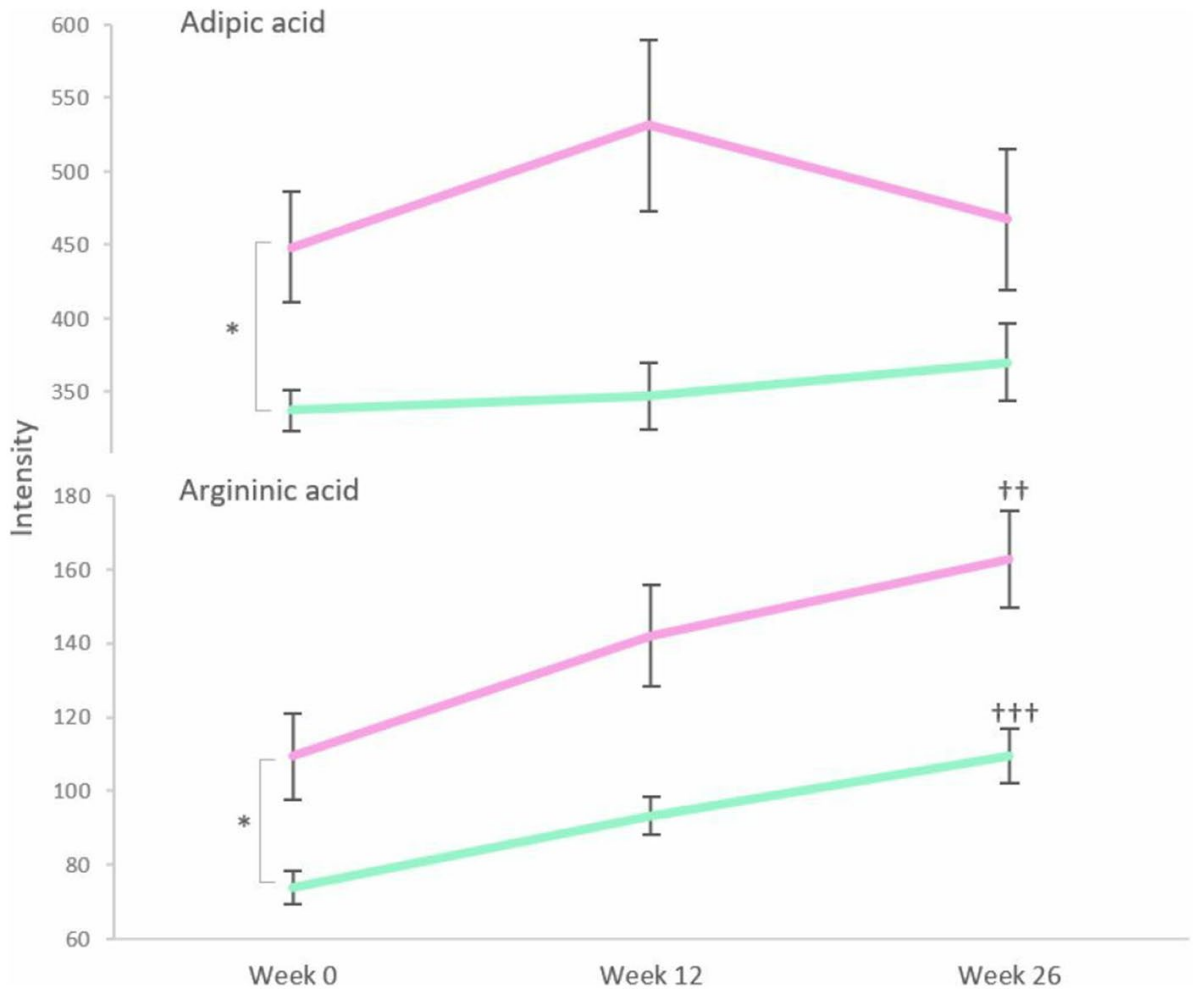
Levels of adipic acid and argininic acid for responders (green) and non-responders (pink) at baseline, week 12 and week 26. Error bars represent SEM. *p < 0.05 between responders and non-responders at baseline. ††*p* < 0.01 and †††*p* < 0.0001 are changes from baseline to week 26 within each group over the time of intervention.

Levels of adipic acid and argininic acid for the responders and non-responders at baseline, week 12, and week 26 are depicted in Figure 5. The measured intensities of adipic acid and argininic acid at baseline were lower for responders than for non-responders (Figure 5, *p* = 0.001 and *p* = 0.0002, respectively). The level of adipic acid remained stable over time in both groups (responders *p* = 0.212; non-responders *p* = 0.696), whereas argininic acid increased by 48% in both groups (responders *p* = 0.00001; non-responders *p* = 0.003).

### Dietary intake and compliance with the NND

There were no differences in the dietary intake between responders and non-responders at baseline after the 1 week run-in period. Dietary intake changes during the intervention are shown in Table 3. As expected, both groups followed the same pattern of eating more fiber and polyunsaturated fat in addition to less saturated fat and added sugar. The mean (±SD) compliance to the NND evaluated by the dietitians was 4.46 ± 0.56 for all subjects (4.56 ± 0.55 and 4.27 ± 0.55 for the responders and non-responders, respectively, *p* = 0.052).

**Table 3:** Changes in energy intake, energy density, and macronutrient intake from weeks 0 to 26 on the basis of the individual 3 day weighed dietary records for responders and non-responders.

	Responders (*n* = 46)	Non-responders (*n* = 24)	*p*-values^a^
Estimated energy requirement, MJ/d^b^	10.7 ± 1.6	11.1 ± 1.8	0.31
Energy intake, kJ/d	−1863 ± 2016***	−764 ± 4,058	0.17
Energy density, kJ/100 g	−87.7 ± 113.3***	−61.8 ± 111.7*	0.42
Protein, % of energy	1.7 ± 2.7***	0.9 ± 3.1	0.33
Carbohydrate, % of energy	0.4 ± 6.9	1.8 ± 7.1	0.45
Fiber, g/10 MJ	7.6 ± 9.7***	6.4 ± 8.6**	0.65
Added sugar, % of energy	−1.9 ± 2.7***	−1.4 ± 3.9	0.54
Total fat, % of energy	−3.3 ± 5.8**	−3.2 ± 5.9*	0.96
SFA, % of energy	−5.2 ± 2.7***	−4.4 ± 3.6***	0.32
MUFA, % of energy	−1.5 ± 4.1*	−2.0 ± 3.6*	0.66
PUFA, % of energy	2.6 ± 2.3***	1.9 ± 2.6**	0.32

Data are presented as mean ± standard deviation. *p < 0.05, **p < 0.005, and ***p < 0.0005.

a Between the responders and non-responders by using Student’s *t* test.

b On the basis of weight and height at baseline by using the Schofield equation, and multiplied by a physical activity level of 1.5.

### Differences between responders and non-responders during follow-up

**Figure 6:**
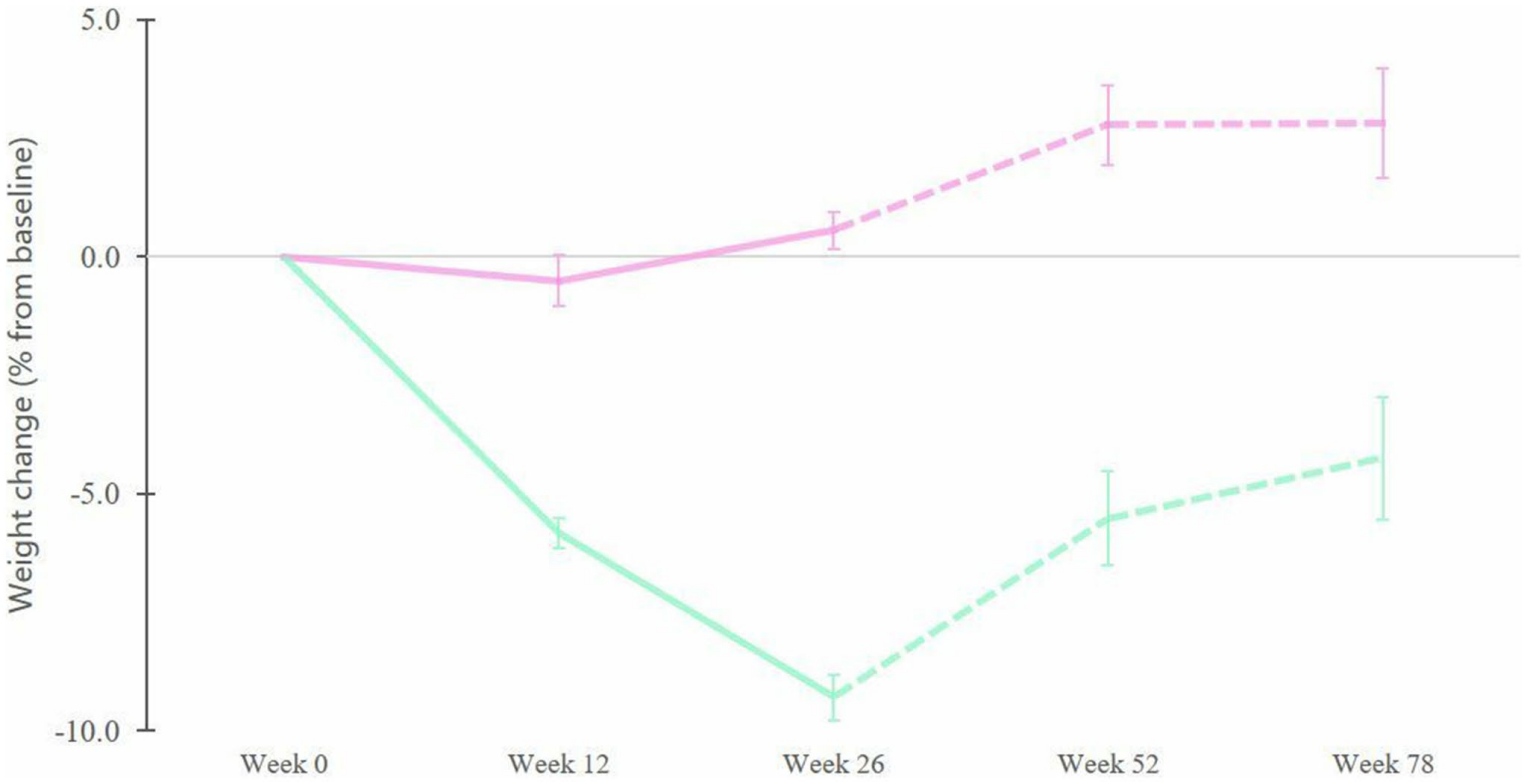
Changes in body weight (in percent from initial weight) for the responders (green) and the non-responders (pink) during 26 weeks of intervention (full line) followed by an additional 52 week follow-up period (dashed line). Error bars represent SEM.

The difference in weight loss between responders and non-responders was significant even before the end of the intervention (i.e., at week 12) and remained significant throughout the follow-up (Figure 6, week 12: *p* < 0.00001, week 26: *p* < 0.00001, week 52: *p* < 0.00001, week 78: *p* = 0.002). Non-responders had no significant change in body weight during the intervention period (26 weeks) but experienced a significant weight gain at 6 and 12 months of follow-up (from 0.6 to 2.8% weight gain, *p* = 0.03 and *p* = 0.004, respectively). Overall, at the end of the follow-up period, responders maintained a weight loss of 4.3% (*p* = 0.002) compared to baseline, whereas non-responders had a weight gain of 2.8% (*p* = 0.03). The difference between the responders and non-responders was 7.1% (*p* = 0.002) by the end of the 26 week intervention and this was maintained throughout the following 52 week follow-up period (Figure 6).

## Discussion

In this study, we investigated the feasibility of predicting weight loss after 26 weeks of NND consumed *ad libitum* among Danish subjects with obesity. In the original trial, a significant weight loss difference of 3.2 kg was found between the NND and ADD groups, and the average weight loss in the NDD group was 4.7 kg (22). Nevertheless, individuals varied widely in their responses, from losing >15% of initial body weight to gaining weight. This is in line with observations from other randomized controlled trials investigating the weight loss efficacy of different diets (11, 12). When combining the clinical baseline data with untargeted metabolomics urine and blood baseline data, QLattice – a symbolic regression machine learning tool – was able to identify a model with two metabolites, urinary levels of adipic acid and argininic acid, that in combination provided a predictive signature for discriminating weight loss responders and non-responders at baseline. Lower levels of both metabolites in urine predicted greater weight loss success following the NND, and this was also observed at 12 weeks. Such a model may serve as a biomarker signature and might be used to optimize weight loss success and is and could be a central step applying precision nutrition in practice.

### Adipic acid and its potential involvement in body weight regulation

Adipic acid (C_6_H_10_O_4_) is a medium-chain fatty acid formed during the β-oxidation of longer-chain dicarboxylic acids derived from ω-oxidation of fatty acids with the same chain length in the microsomes (41). The β-oxidation of the longer dicarboxylic acids takes place in mitochondria and peroxisomes with the end product being succinyl-coenzyme A (CoA), which can enter the tricarboxylic acid cycle (TCA) and produce succinate (41). Of the dicarboxylic acids, adipic acid seems to be the major metabolic end-product (42) and is known to be present in the blood circulation, but has also been detected as intestinal metabolite (43).

Studies have shown that levels of dicarboxylic acids – including adipic acid – are increased in patients who are ketotic due to diabetes or in those with congenital defects in fatty acid metabolism; adipic acid levels also tend to be higher in non-ketotic diabetes, but not significantly (42). There were no subjects with diabetes included in our study, however, some of the participants had prediabetes both among the responders and the non-responders. Within each group, there was no difference in weight loss between those with normal glycemic status (NGS) and those with prediabetes, but those with prediabetes had a greater decrease in fasting blood glucose following the NND no matter if they were responders (prediabetes −0.5 ± 0.3 mmol/L and NGS −0.2 ± 0.3 mmol/L, *p* = 0.004) or non-responders (prediabetes: −0.8 ± 0.7 mmol/L and NGS 0.0 ± 0.3 mmol/L, *p* = 0.0009). Accordingly, the high-fiber NND had a greater effect on glycemic control among those with prediabetes independent of weight loss, which corroborates our previous reports (44).

Higher levels of fecal adipic acid and other TCA cycle intermediates in the gut have been observed in Chinese subjects with obesity compared to subjects with normal weight (43). It has been speculated that the Prevotella enterotype – where the *Prevotella* spp. (P) are in higher abundance than the *Bacteroides spp*. (B) – promotes weight loss as a result of increased appetite-suppressing hormones such as glucagon-like peptide-1 (GLP-1) and peptide YY (PYY) through the production of propionate on a diet rich in fiber (45). In a subset of the subjects (*n* = 18), fecal samples were earlier analyzed (46) from which 80% of the non-responders’ enterotype was dominated by a low P/B-ratio, whereas 62% of the responders’ enterotype was dominated by a high P/B-ratio. This might partly explain why the responders had better weight loss on the NND compared to the non-responders. These preliminary results add to a growing body of literature about the important role of the gut microbiome in body weight homeostasis under certain dietary regimens, but more studies are needed to establish a comprehensive understanding of the impact of intestinal microbiota on weight loss responses.

Lastly, it should be mentioned that adipic acid is a possible environmental contaminant used in the food industry and metabolized by humans to some extent into glutamic, lactic, beta-ketoadipic, and citric acids. Any unmetabolized adipic acid is excreted in the urine (47, 48). Levels of adipic acid did not change over our 26 week dietary intervention period, which indicates that differences in urinary adipic acid between responders and non-responders likely result from differences in the inherent metabolic process and not from differences in dietary intake, and thus also not from differences in habitual diets between groups.

### Argininic acid and its potential involvement in body weight regulation

Argininic acid (C_6_H_13_N_3_O_3_) is a by-product of the urea cycle. It has been proposed that arginine is converted to α-keto-δ-guanidinovaleric acid *in vivo* by transamination and further converted to argininic acid upon hydrogenation (49, 50). Tissue accumulation of argininic acid occurs in patients with hyperargininemia. Here, levels of guanidinosuccinate are decreased and α-keto-δ-guanidinovaleric and argininic acid concentrations are increased (51). Nevertheless, it has also been shown that argininic acid is hydrolyzed by arginase in the liver to produce urea (52). The responders had lower levels of argininic acid, α-keto-δ-guanidinovaleric as well as creatinine compared to the non-responders at baseline, which collectively suggests they also had lower levels of arginine. This, in turn, suggests a somewhat upregulated urea cycle, as impaired urea cycle flux leads to hyperargininemia (i.e., higher circulating arginine) (53). Urea cycle is an energy consuming process hence these results indicate that responders may be more primed for increased energy turnover and therefore greater energy expenditure than non-responders. This would promote a more negative energy balance in response to dieting and consequently, more weight loss.

At baseline, the responders and non-responders were comparable, with the exception that responders were older than non-responders. Interestingly, previous studies have also found that age is a determinant of weight loss success, with older individuals typically losing more weight than younger individuals after the same treatment (54–56). It is not entirely clear if the mechanism behind this observation is biological, or behavioral (e.g., better compliance). The levels of argininic acid in our study correlated with the weight change even after adjusting for age, so we can rule out that argininic acid is simply a biomarker for older age-related greater weight loss.

The almost 50% increase in the urinary levels of argininic acid in both groups after the intervention suggests that this increase is likely not a direct result of weight change, but more likely related to features of the NND *per se*. Both groups had a greater intake of protein throughout the intervention with no difference between them (*p* = 0.33) which may partly explain the increased levels since meat is a good source of arginine (57). Also, one of the characteristics of the NND is a greater intake of nuts (22, 24), which are also a good food source of arginine (58). This hypothesis is supported by the finding that experimental supplementation with creatine increases argininic acid and α-keto-δ-guanidinovaleric (59). Thus these dietary factors may help explain the increase in the levels of argininic acid after the NDD in both groups.

### Compliance, weight maintenance, and model validation

Overall compliance with the NND was high, but was somewhat higher among the responders than the non-responders. As observed in previous studies (9–11), adherence to any diet is important for weight loss as it builds on good habits, but compliance with calorie restriction remains the most important factor. In an earlier paper, we investigated dietary compliance with the NND based on the patterns of urinary metabolites (60). A number of subjects were found to be non-compliant, however, in the present study, we did not observe any direct link between compliance and extent of weight loss, as the “non-compliant” subjects were equally distributed between our two groups. Among subjects who were misclassified by the prediction model, three of them showed high levels of NND food biomarkers in their urine and would be seen as being highly compliant, nevertheless, the current model predicted them to be non-responders. This underlines that factors other than compliance affect the individual response to the NND.

Studies often report short-term weight loss success after a variety of diet interventions, but in most cases, subjects tend to regain the lost weight after the intervention (61). In our study, we found that responders, having lost ≥5% of their initial weight after 26 weeks on the NND, were also able to maintain greater weight loss than non-responders 1 year later even though both groups regained some weight. We also observed that the subjects’ weight loss success was already evident after 12 weeks on the NND and could be predicted by the model, indicating that the effect was robust and internally valid at both earlier and later time points. This supports the observation from other studies where early weight loss has been a good predictor for later weight loss success (62). We also tested if the model could predict the weight changes among subjects following the ADD within the SHOPUS study (results not shown). The model did not perform well in that scenario, potentially due to lack of power but it might also be that the model is diet-specific and not a general weight loss prediction model.

### Strengths and limitations of the study

The cut-offs to classify responders and non-responders were chosen arbitrarily to ensure a clear separation of the groups. Nevertheless, a weight loss of ≥5% is normally considered a clinically significant weight loss (23) whereas a cut-off of <2% is normally used to confirm weight stability (63). The use of the different – but complementary – analytical platforms and the use of both blood and urine samples from each subject is an advantage of the study, as no single approach is capable of capturing the phenotypic complexity of human metabolic profile (64). However, in retrospect, it can be questioned whether one analytical platform could have been sufficient as both metabolites came from the LC–MS dataset even though all data were included in the model development. This could not have been known beforehand, which is why all available data from all analytical platforms were included in our analysis. Our subjects were thus deeply phenotyped but the low sample size should be carefully considered when working with thousands of individual variables for effective data integration and machine learning (65). Even though we built an internally robust method and used a separate test set, we cannot rule out that overfitting may have occurred. It should be noted that the adipic acid and argininic acid levels are relative intensities and not absolute values that can be used in clinical settings. For example, argininic acid increased in intensity after the NND in both groups, which in real-life settings can result in some non-responders being misclassified as responders if they habitually follow an NND-like diet.

In the present study, we were able to identify a simple model based on baseline data predicting the likelihood of achieving a clinically significant weight loss on an *ad libitum* NND using an untargeted multi-platform metabolomics and machine learning approach. Such models can be used to optimize precision dietary therapies for the treatment of obesity and are a central step in applying precision nutrition in practice. Understanding the predictive features of the weight loss response will help elucidate the interplay between metabolic processes, diet, and individual susceptibility and behaviour. However, there is a need to investigate similar datasets to evaluate whether the current findings may be generalized to other weight loss diets.

## Data availability statement

The raw data supporting the conclusions of this article will be made available by the authors, without undue reservation.

## Ethics statement

The studies involving human participants were reviewed and approved by the ethics committee of the Capital Region of Denmark. The ethics committee waived the requirement of written informed consent for participation.

## Author contributions

AA was investigator in the original trial. KP, LD, and MH contributed to conception and design of the study. LD was responsible for the LC–MS analysis. KP analyzed and annotated the LC–MS data, responsible for statistical analysis, model development supervised by VS-L and SD and wrote the first draft of the manuscript. AT, BK, and SE were responsible for the NMR analysis. AT annotated the NMR data. FM and LD reviewed, edited the manuscript, and provided supervision. All authors contributed to the article and approved the submitted version.

## Funding

The study was funded by The Nordea Foundation Denmark, a PhD scholarship from the King Saud bin Abdulaziz University for Health Sciences via The Saudi Arabian Cultural Office, and Novo-Nordisk Foundation (NNF19OC0056246).

## Conflict of interest

VS-L and SD are employed at Abzu, developers of the QLattice®.

The remaining authors declare that the research was conducted in the absence of any commercial or financial relationships that could be construed as a potential conflict of interest.

## Publisher’s note

All claims expressed in this article are solely those of the authors and do not necessarily represent those of their affiliated organizations, or those of the publisher, the editors and the reviewers. Any product that may be evaluated in this article, or claim that may be made by its manufacturer, is not guaranteed or endorsed by the publisher.

## References

[1] Blüher M. Obesity: global epidemiology and pathogenesis. Nat Rev Endocrinol. (2019) 15:288–98. doi: 10.1038/s41574-019-0176-8 30814686

[2] Kahn SE Hull RL Utzschneider KM. Mechanisms linking obesity to insulin resistance and type 2 diabetes. Nature. (2006) 444:840–6. doi: 10.1038/nature05482 17167471

[3] Powell-Wiley TM Poirier P Burke LE Després JP Gordon-Larsen P Lavie CJ. Obesity and cardiovascular disease a scientific statement from the American Heart Association. Circulation. (2021) 143:E984–E1010. doi: 10.1161/CIR.0000000000000973, PMID: 33882682 PMC8493650

[4] Polyzos SA Kountouras J Mantzoros CS. Obesity and nonalcoholic fatty liver disease: from pathophysiology to therapeutics. Metabolism. (2019) 92:82–97. doi: 10.1016/j.metabol.2018.11.014 30502373

[5] Calle E Rodriguez C Walker-thurmond K Overweight TM. Obesity, and mortality from Cancer in a prospectively studied cohort of U.S. Adults N Engl J Med. (2003) 348:1625–38. doi: 10.1056/NEJMoa021423, PMID: 12711737

[6] Bray GA. The Battle of the bulge: A history of obesity research. Pittsburgh, PA: Dorranc ePublishing Co., Inc. (2007).

[7] Yancy WS Westman EC McDuffie JR Grambow SC Jeffreys AS Bolton J. A randomized trial of a low-carbohydrate diet vs orlistat plus a low-fat diet for weight loss. Arch Intern Med. (2010) 170:136–45. doi: 10.1001/archinternmed.2009.492, PMID: 20101008

[8] Gardner CD Kiazand A Kim S Stafford RS Balise RR Kraemer HC. Comparison of the Atkins, zone, Ornish, and LEARN diets for change in weight and related risk factors among Overweight premenopausal women. The a TO Z weight loss study: a randomized trial. J Am Med Assoc. (2007) 297:969–77. doi: 10.1001/jama.297.9.969, PMID: 17341711

[9] Dansinger ML Gleason JA Griffith JL Selker HP Schaefer EJ. Comparison of the Atkins, Ornish, weight watchers, and zone diets for weight loss and heart disease risk reduction: a randomized trial. J Am Med Assoc. (2005) 293:43–53. doi: 10.1001/jama.293.1.43, PMID: 15632335

[10] Sacks FM Bray GA Carey VJ Smith SR Ryan DH Anton SD. Comparison of weight-loss diets with different compositions of fat, protein, and carbohydrates. N Engl J Med. (2009) 360:859–73. doi: 10.1056/NEJMoa0804748, PMID: 19246357 PMC2763382

[11] Greenberg I Stampfer MJ Schwarzfuchs D Shai I. Adherence and success in long-term weight loss diets: the dietary intervention randomized controlled trial (direct). J Am Coll Nutr. (2009) 28:159–68. doi: 10.1080/07315724.2009.10719767, PMID: 19828901

[12] Bray GA Ryan DH Johnson W Champagne CM Johnson CM Rood J. Markers of dietary protein intake are associated with successful weight loss in the POUNDS lost trial. Clin Obes. (2017) 7:166–75. doi: 10.1111/cob.12188, PMID: 28340516 PMC5517018

[13] Lean M Brosnahan N McLoone P McCombie L Higgs AB Ross H. Feasibility and indicative results from a 12-month low-energy liquid diet treatment and maintenance programme for severe obesity. Br J Gen Pract. (2013) 63:e115–24. doi: 10.3399/bjgp13X663073 23561690 PMC3553637

[14] Pigsborg K Magkos F. Metabotyping for precision nutrition and weight management: hype or Hope? Curr Nutr Rep. (2022) 11:117–23. doi: 10.1007/s13668-021-00392-y 35025088

[15] Dragsted LO. The metabolic nature of individuality. Nat Food. (2020) 1:327–8. doi: 10.1038/s43016-020-0104-z 37128096

[16] Fiehn O. Metabolomics – the link between genotypes and phenotypes. Plant Mol Biol. (2002) 48:155–71. doi: 10.1023/A:1013713905833 11860207

[17] Rochfort S. Biology and implications for natural products research. J Nat Prod. (2005) 68:1813–20. doi: 10.1021/np050255w, PMID: 16378385

[18] Hjorth MF Zohar Y Hill JO Astrup A. Personalized dietary management of overweight and obesity based on measures of insulin and glucose. Annu Rev Nutr. (2018) 38:245–72. doi: 10.1146/annurev-nutr-082117-051606, PMID: 29856931 PMC9105825

[19] Wilstrup C Kasak J. Symbolic regression outperforms other models for small data sets (2021) arXiv [Preprint]. 1–10 doi: 10.48550/arXiv.2103.15147

[20] Wenninger S Kaymakci C Wiethe C. Explainable long-term building energy consumption prediction using QLattice. Appl Energy. (2021) 308:118300. doi: 10.1016/j.apenergy.2021.118300

[21] Christensen NJ Demharter S MacHado M Pedersen L Salvatore M Stentoft-Hansen V. Identifying interactions in omics data for clinical biomarker discovery using symbolic regression. Bioinformatics. (2022) 38:3749–58. doi: 10.1093/bioinformatics/btac405, PMID: 35731214 PMC9344843

[22] Poulsen SK Due A Jordy AB Kiens B Stark KD Stender S. Health effect of the new nordic diet in adults with increased waist circumference: a 6-mo randomized controlled trial. Am J Clin Nutr. (2014) 99:35–45. doi: 10.3945/ajcn.113.069393, PMID: 24257725

[23] Williamson DA Bray GA Ryan DH. Is 5% weight loss a satisfactory criterion to define clinically significant weight loss? Obesity. (2015) 23:2319–20. doi: 10.1002/oby.21358, PMID: 26523739

[24] Mithril C Dragsted LO Meyer C Blauert E Holt MK Astrup A. Guidelines for the new Nordic diet. Public Health Nutr. (2012) 15:1941–7. doi: 10.1017/S136898001100351X, PMID: 22251407

[25] Matthews DR Hosker JP Rudenski AS Naylor BA Treacher DF Turner RC. Homeostasis model assessment: insulin resistance and β-cell function from fasting plasma glucose and insulin concentrations in man. Diabetologia. (1985) 28:412–9. doi: 10.1007/BF00280883, PMID: 3899825

[26] Matsuda M DeFronzo RA. Insulin sensitivity indices obtained from oral glucose tolerance testing: comparison with the euglycemic insulin clamp. Diabetes Care. (1999) 22:1462–70. doi: 10.2337/diacare.22.9.1462, PMID: 10480510

[27] Poulsen SK Crone C Astrup A Larsen TM. Long-term adherence to the new Nordic diet and the effects on body weight, anthropometry and blood pressure: a 12-month follow-up study. Eur J Nutr. (2015) 54:67–76. doi: 10.1007/s00394-014-0686-z, PMID: 24664189

[28] Barri T Holmer-Jensen J Hermansen K Dragsted LO. Metabolic fingerprinting of high-fat plasma samples processed by centrifugation- and filtration-based protein precipitation delineates significant differences in metabolite information coverage. Anal Chim Acta. (2012) 718:47–57. doi: 10.1016/j.aca.2011.12.065, PMID: 22305897

[29] Gürdeniz G Kristensen M Skov T Dragsted LO. The effect of LC-MS data preprocessing methods on the selection of plasma biomarkers in fed vs. fasted rats. Meta. (2012) 2:77–99. doi: 10.3390/metabo2010077, PMID: 24957369 PMC3901197

[30] Smith CA Want EJ O’Maille G Abagyan R Siuzdak G. XCMS: processing mass spectrometry data for metabolite profiling using nonlinear peak alignment, matching, and identification. Anal Chem. (2006) 78:779–87. doi: 10.1021/ac051437y, PMID: 16448051

[31] Kuhl C Tautenhahn R Böttcher C Larson TR Neumann S. CAMERA: an integrated strategy for compound spectra extraction and annotation of liquid chromatography/mass spectrometry data sets. Anal Chem. (2012) 84:283–9. doi: 10.1021/ac202450g, PMID: 22111785 PMC3658281

[32] Sumner LW Reily MD Higashi R Nicholls AW Marriott P Hardy N. Proposed minimum reporting standards for chemical analysis. Metabolomics. (2007) 3:211–21. doi: 10.1007/s11306-007-0082-2, PMID: 24039616 PMC3772505

[33] Trimigno A Khakimov B Rasmussen MA Dragsted LO Larsen TM Astrup A. Human blood plasma biomarkers of diet and weight loss among centrally obese subjects in a new Nordic diet intervention. Front Nutr. (2023) 10:1198531. doi: 10.3389/fnut.2023.1198531, PMID: 37396134 PMC10308042

[34] Trimigno A Khakimov B Savorani F Poulsen SK Astrup A Dragsted LO. Human urine 1H NMR metabolomics reveals alterations of the protein and carbohydrate metabolism when comparing habitual average Danish diet vs. healthy new Nordic diet. Nutrition. (2020) 79–80:110867. doi: 10.1016/j.nut.2020.110867, PMID: 32619792

[35] Khakimov B Mobaraki N Trimigno A Aru V Engelsen SB. Signature mapping (SigMa): an efficient approach for processing complex human urine 1H NMR metabolomics data. Anal Chim Acta. (2020) 1108:142–51. doi: 10.1016/j.aca.2020.02.025, PMID: 32222235

[36] Savorani F Tomasi G Engelsen SB. Icoshift: a versatile tool for the rapid alignment of 1D NMR spectra. J Magn Reson. (2010) 202:190–202. doi: 10.1016/j.jmr.2009.11.012, PMID: 20004603

[37] Lawton WH Sylvestre EA. Self modeling curve resolution. Technometrics. (1971) 13:617–33. doi: 10.1080/00401706.1971.10488823

[38] Engelsen SB Savorani F Rasmussen MA. Chemometric exploration of quantitative NMR data. eMagRes. (2013) 2:267–78. doi: 10.1002/9780470034590.emrstm1304

[39] Abzu. feyn 3.0.4. (Accessed November 17, 2022). Available at: https://pypi.org/project/feyn/

[40] Wilstrup C Cave C. Combining symbolic regression with the cox proportional hazards model improves prediction of heart failure deaths. BMC Med Inform Decis Mak. (2022) 22:1–7. doi: 10.1186/s12911-022-01943-1 35879758 PMC9316394

[41] Mingrone G Castagneto-Gissey L Macé K. Use of dicarboxylic acids in type 2 diabetes. Br J Clin Pharmacol. (2013) 75:671–6. doi: 10.1111/j.1365-2125.2012.04177.x, PMID: 22242741 PMC3575934

[42] Yoshioka K Shimojo N Nakanishi T Naka K Okuda K. Measurements of urinary adipic acid and suberic acid using high-performance liquid chromatography. J Chromatogr B Biomed Sci Appl. (1994) 655:189–93. doi: 10.1016/0378-4347(94)80022-7, PMID: 8081464

[43] Wan Y Yuan J Li J Li H Yin K Wang F. Overweight and underweight status are linked to specific gut microbiota and intestinal tricarboxylic acid cycle intermediates. Clin Nutr. (2020) 39:3189–98. doi: 10.1016/j.clnu.2020.02.014, PMID: 32164980

[44] Mao T Huang F Zhu X Wei D Chen L. Effects of dietary fiber on glycemic control and insulin sensitivity in patients with type 2 diabetes: a systematic review and meta-analysis. J Funct Foods. (2021) 82:104500. doi: 10.1016/j.jff.2021.104500

[45] Christensen L Roager HM Astrup A Hjorth MF. Microbial enterotypes in personalized nutrition and obesity management. Am J Clin Nutr. (2018) 108:645–51. doi: 10.1093/ajcn/nqy175, PMID: 30239555

[46] Hjorth MF Roager HM Larsen TM Poulsen SK Licht TR Bahl MI. Pre-treatment microbial Prevotella-to-Bacteroides ratio, determines body fat loss success during a 6-month randomized controlled diet intervention. Int J Obes. (2018) 42:580–3. doi: 10.1038/ijo.2017.220, PMID: 28883543 PMC5880576

[47] Luttrell WE Klaassen GR. Adipic acid. J Chem Heal Saf. (2016) 23:44–6. doi: 10.1016/j.jchas.2016.05.005

[48] Rusoff II Baldwin RR Domingues FJ Monder C Ohan WJ Thiessen R. Intermediary metabolism of adipic acid. Toxicol Appl Pharmacol. (1960) 2:316–30. doi: 10.1016/0041-008X(60)90060-0 14440268

[49] Cooper AJL Meister A. Cyclic forms of the α-keto acid analogs of arginine, citrulline, homoarginine, and homocitrulline. J Biol Chem. (1978) 253:5407–10. doi: 10.1016/S0021-9258(17)30386-1, PMID: 670205

[50] Marescau B De DPP Lowenthal A Qureshi IA Antonozzi I Bachmann C. Guanidino compound analysis as a complementary diagnostic parameter for hyperargininemia: follow-up of Guanidino compound levels during therapy. Pediatr Res. (1990) 27:297–303. doi: 10.1203/00006450-199003000-00020 1690873

[51] Marescau B Lowenthal A. Isolation and identification of some guanidino compounds in the urine of patients with hyperargininaemia by liquid chromatography, thin-layer chromatography and gas chromatography-mass spectrometry. J Chromatogr. (1981) 224:185–95. doi: 10.1016/S0378-4347(00)80156-5

[52] Calvery HO Block WD. The specificity of the enzyme arginase. J Biol Chem. (1934) 107:155–60. doi: 10.1016/S0021-9258(18)75395-7

[53] Scaglia F Lee B. Clinical, biochemical, and molecular spectrum of hyperargininemia due to arginase I deficiency. Am J Med Genet – Semin Med Genet. (2006) 142C:113. doi: 10.1002/ajmg.c.30091 PMC4052756 16602094

[54] Finkler E Heymsfield SB St-Onge MP. Rate of weight loss can be predicted by patient characteristics and intervention strategies. J Acad Nutr Diet. (2012) 112:75–80. doi: 10.1016/j.jada.2011.08.034, PMID: 22717178 PMC3447534

[55] DeLuca L Toro-Ramos T Michaelides A Seng E Swencionis C. Relationship between age and weight loss in noom: quasi-experimental study. JMIR Diabetes. (2020) 5:e18363. doi: 10.2196/18363 32497017 PMC7303833

[56] Svetkey LP Clark JM Funk K Corsino L Batch BC Hollis JF. Greater weight loss with increasing age in the weight loss maintenance trial. Obesity. (2014) 22:39–44. doi: 10.1002/oby.20506, PMID: 23640912 PMC3849225

[57] Wu G Bazer FW Davis TA Kim SW Li P Rhoads JM. Arginine metabolism and nutrition in growth, health and disease Guoyao. Amino Acids. (2009) 37:153–68. doi: 10.1007/s00726-008-0210-y, PMID: 19030957 PMC2677116

[58] Brufau G Boatella J Rafecas M. Nuts: source of energy and macronutrients. Br J Nutr. (2006) 96:S24–8. doi: 10.1017/BJN20061860 17125529

[59] Derave W Marescau B Vanden Eede E Eijnde BO De Deyn PP Hespel P. Plasma guanidino compounds are altered by oral creatine supplementation in healthy humans. J Appl Physiol. (2004) 97:852–7. doi: 10.1152/japplphysiol.00206.2004, PMID: 15107411

[60] Andersen M-BS Rinnan Å Manach C Poulsen SK Pujos-Guillot E Larsen TM. Untargeted metabolomics as a screening tool for estimating compliance to a dietary pattern. J Proteome Res. (2014) 13:1405–18. doi: 10.1021/pr400964s, PMID: 24444418

[61] Franz MJ VanWormer JJ Crain AL Boucher JL Histon T Caplan W. Weight-loss outcomes: a systematic review and Meta-analysis of weight-loss clinical trials with a minimum 1-year follow-up. J Am Diet Assoc. (2007) 107:1755–67. doi: 10.1016/j.jada.2007.07.017, PMID: 17904936

[62] James BL Roe LS Loken E Rolls BJ. Early predictors of weight loss in a 1-year behavioural weight-loss programme. Obes Sci Pract. (2018) 4:20–8. doi: 10.1002/osp4.149, PMID: 29479461 PMC5818734

[63] Magkos F Fraterrigo G Yoshino J Luecking C Kirbach K Kelly SC. Effects of moderate and subsequent progressive weight loss on metabolic function and adipose tissue biology in humans with obesity. Cell Metab. (2016) 23:591–601. doi: 10.1016/j.cmet.2016.02.005, PMID: 26916363 PMC4833627

[64] Zhang A Sun H Wang X. Power of metabolomics in biomarker discovery and mining mechanisms of obesity. Obes Rev. (2013) 14:344–9. doi: 10.1111/obr.12011, PMID: 23279162

[65] Smilde AK Næs T Liland KH. Multiblock data fusion in statistics and machine learning - applications in the natural and life sciences. Chichester, West Sussex, UK: John Wiley & Sons Ltd (2022).

