# Supplementary material for: Predicting weight loss success on a new Nordic diet: an untargeted multi-platform metabolomics and machine learning approach
Source: Front Nutr. 2023 Aug 1;10:1191944. doi: 10.3389/fnut.2023.1191944 (PMC10434509; doi:10.3389/fnut.2023.1191944)
Supplement: Supplementary file 1 [file Data_Sheet_1.docx]

Supplementary Material

Predicting weight loss success on a New Nordic Diet: an untargeted multi-platform metabolomics and machine learning approach

Kristina Pigsborg^1*^, Valdemar Stentoft-Larsen^2^, Samuel Demharter^2^, Mona Adnan Aldubayan^1,3^, Alessia Trimigno^4^, Bekzod Khakimov^4^, Søren Balling Engelsen^4^, Arne Astrup^5^, Mads Fiil Hjorth^5^, Lars Ove Dragsted^1^, Faidon Magkos^1^

^1^Department of Nutrition, Exercise and Sports, University of Copenhagen, Frederiksberg, Denmark

^2^Abzu ApS, Copenhagen, Denmark

^3^King Saud bin Abdulaziz University for Health Sciences, College of Applied Medical Sciences, Riyadh, Saudi Arabia

^4^Department of Food Science, University of Copenhagen, Frederiksberg, Denmark

^5^Obesity and Nutritional Sciences, Novo Nordisk Foundation, Hellerup, Denmark

*** Correspondence:**Kristina Pigsborg
kpj@nexs.ku.dk


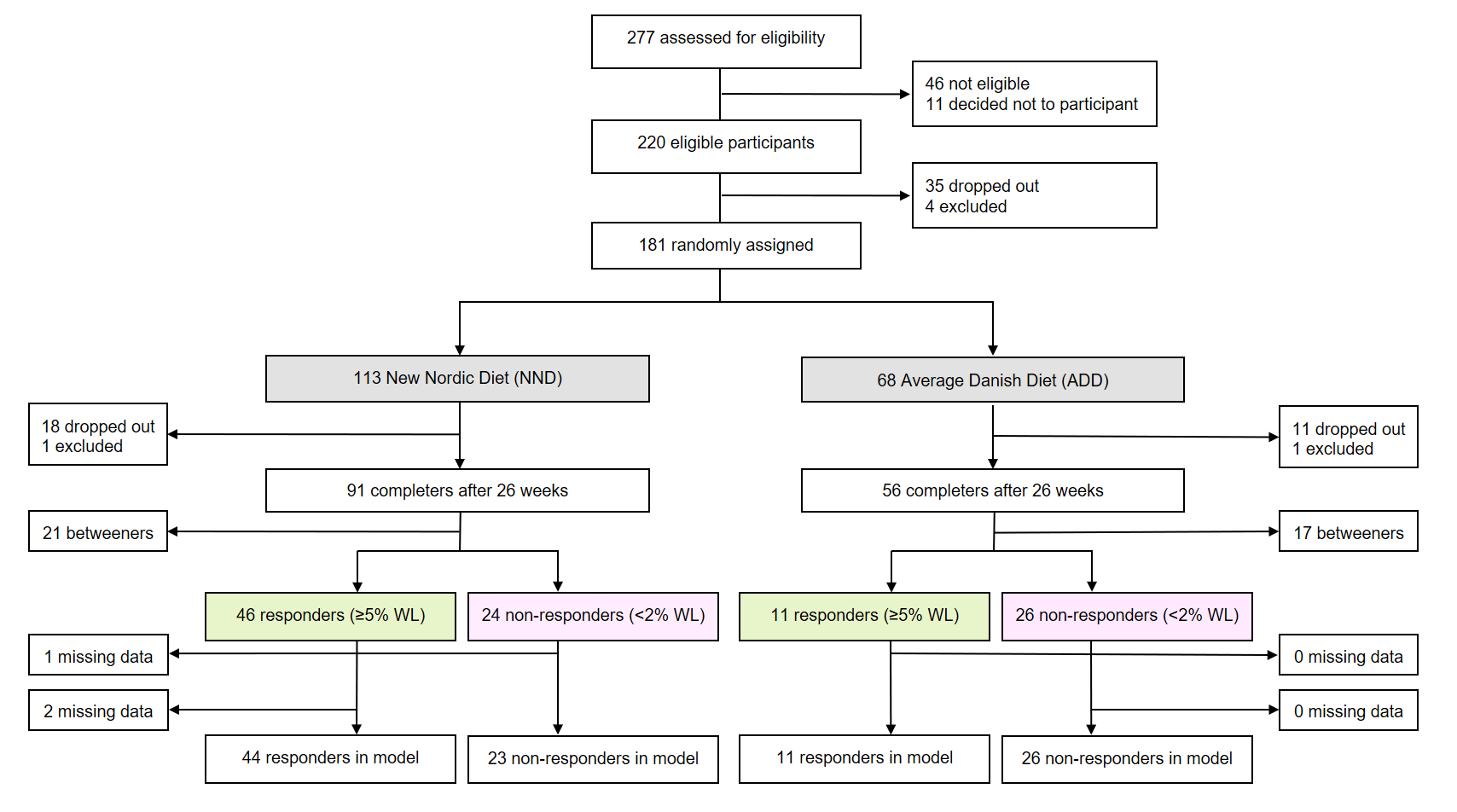


**Supplementary Figure 1.** Flow diagram of participants in a dietary intervention with the New Nordic Diet or Average Danish Diet.

**Supplementary Figure 2.** Inter-batch variance for the different LC-MS dataset after preprocessing. Points with same color are from the same plate.

**
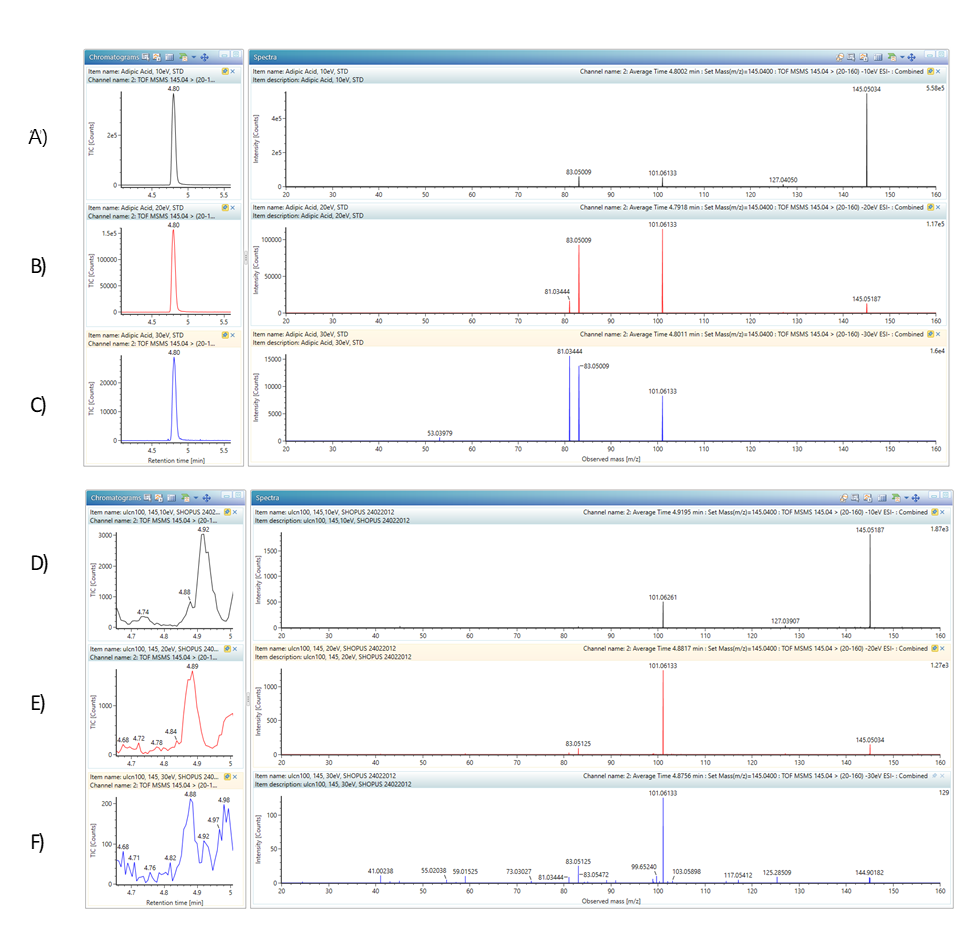
**

**Supplementary Figure 3.** Structure elucidation of adipic acid using MS/MS data. Chromatogram and spectra of adipic acid (CAS: 124-04-9) ionized with 10 (A), 20 (B) and 30 (C) eV and of a random sample from the dataset ionized with 10 (D), 20 (E) and 30 (F) eV showing the similarity of retention time and fragmentation pattern. Please note the 300 times difference in intensity between A, B, C >> D, E, F.


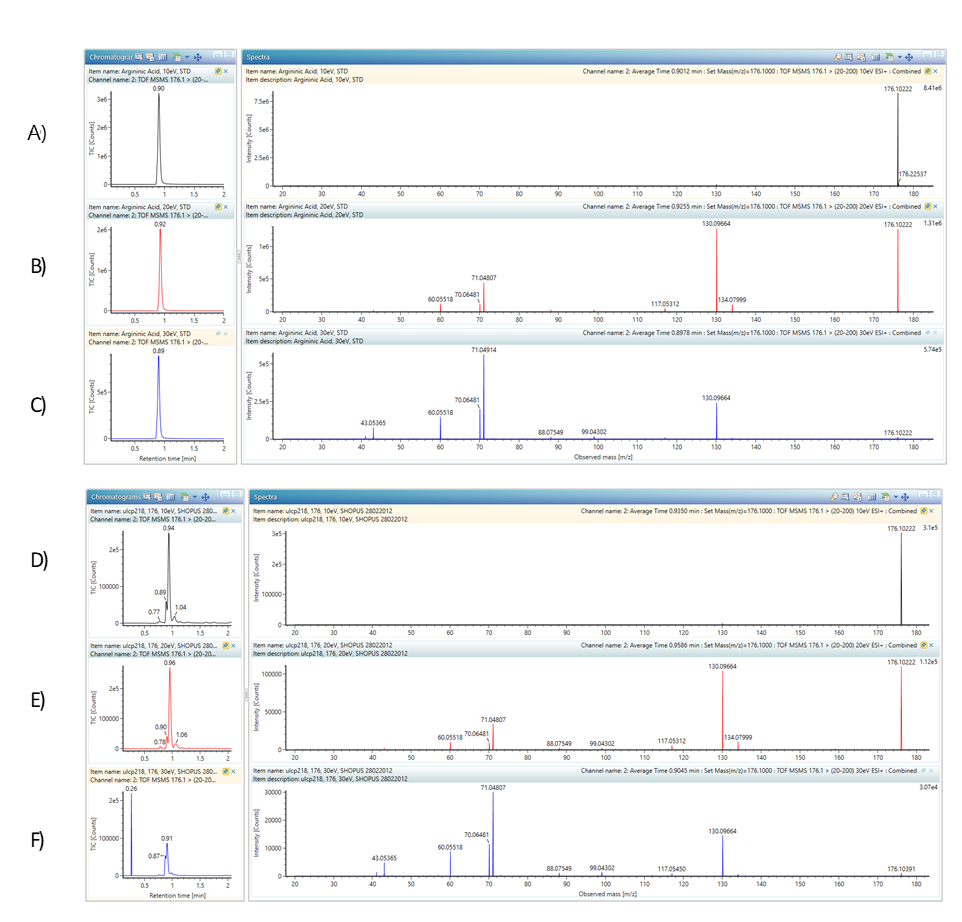


**Supplementary Figure 4.** Structure elucidation of argininic acid using MS/MS data. Chromatogram and spectra of argininic acid (CAS: 157-07-3) ionized with 10 (A), 20 (B) and 30 (C) eV and of a random sample from the dataset ionized with 10 (D), 20 (E) and 30 (F) eV showing the similarity of retention time and fragmentation pattern.

**Supplementary Table 1.** Optimized parameters used for preprocessing of plasma and urine untargeted LC-MS profiling data using R (ver.4.2.1) with the package XCMS. The preprocessing included the following steps: (1) peak picking using the CentWave algorithm that is suitable for centroid data acquisition in case of partially overlapping features, (2) peak grouping using the PeakDensity method, based on the distribution of identified peaks along the RT within slices of overlapping m/z ranges, (3) peak alignment using the PeakGroups method, based on the alignment of features present in most of the samples, (4) repeat peak grouping, with a change of some default parameters, and (5) gap filling using the FillChromPeaks method to estimate the intensity of missing features.

| **Preprocessing steps** | **Algorithm** | **Plasma samples** | **Urine samples** |
| --- | --- | --- | --- |
| (1) Peak picking | CentWave | ppm = 30  peakwidth = c(0.03,0.15)*60 snthresh = 18 noise = 0 prefilter = c(3,15) integrate = 2 mzdiff = -0.001 verboseColumns = TRUE fitgauss = TRUE | ppm = 30  peakwidth = c(0.03,0.15)*60 snthresh = 18 noise = 0 prefilter = c(3,15) integrate = 2 mzdiff = -0.001 verboseColumns = TRUE fitgauss = TRUE |
| (2) Peak grouping | Peakdensity | binSize = 0.01 bw = 0.2*60 minSamples = 3 minFraction = 0.5 maxFeatures = 20 | binSize = 0.01 bw = 0.2*60 minSamples = 3 minFraction = 0.5 maxFeatures = 20 |
| (3) Peak alignment | PeakGroups | smooth = "loess" span = 0.6 minFraction = 0.8 family = "gaussian" extraPeaks = 3 | smooth = "loess" span = 0.6 minFraction = 0.8 family = "gaussian" extraPeaks = 3 |
| (4) Peak grouping | PeakDensity | binSize = 0.01 bw = 0.02*60 minSamples = 4 minFraction = 0.75 maxFeatures = 20 | binSize = 0.01 bw = 0.02*60 minSamples = 4 minFraction = 0.75 maxFeatures = 20 |
| (5) Gap filling | FillChromPeaks | expandMz = 0  expandRt = 0  ppm = 30 | expandMz = 0  expandRt = 0  ppm = 30 |

## Supplementary Table 2. Experimental conditions for UHPLC (Waters Acquity) -coupled tandem mass spectrometry (Vion IMS QTOF mass spectrometer, Waters Corporation, Manchester, UK)

| **Column and pre-column** | A Waters ACQUITY UPLC HSS T3 100 Å, pore size, 1.8 μm particle size, 2.1 mm ø x 100 mm column (Milford, USA) coupled with a pre-column (VanGaurd HSS T3 C18 column (2.1 x 5 mm, 1.8 μm) was used at a temperature of 50 °C for chromatographic separation. | | | | | | |
| --- | --- | --- | --- | --- | --- | --- | --- |
| **Mobile phases** | Five μL of each sample were injected into the graduate mobile phase A (0.1 % formic acid in Milli-Q water), B (100 % methanol), C (1 M ammonium acetate in methanol) and solvent D (100 % isopropanol) over a 10.0 minutes period as presented below: | | | | | | |
|  | Time (min) | Flow Rate (mL min^-1^) | Mobile phase A (%) | Mobile phase B (%) | Mobile phase C (%) | Mobile phase D (%) | Curve |
|  | 0.00 | 0.4 | 100 | 0 | 0 | 0 | Initial |
|  | 0.75 | 0.4 | 100 | 0 | 0 | 0 | 6 |
|  | 6.00 | 0.5 | 0 | 100 | 0 | 0 | 7 |
|  | 6.50 | 0.5 | 0 | 0 | 70 | 30 | 7 |
|  | 8.00 | 0.6 | 0 | 0 | 70 | 30 | 7 |
|  | 8.10 | 0.4 | 0 | 0 | 70 | 30 | 6 |
|  | 9.00 | 0.4 | 100 | 0 | 0 | 0 | 5 |
|  | 10.00 | 0.4 | 100 | 0 | 0 | 0 | 5 |
| **Mass spectrometry** | Mass spectrometry analysis was performed un full scan MS mode in range from 30 to 950 m/z. Scan time was set to 0.1 s for both modes and collision energy and ion-mobility were off. For both modes, ion source temperature was at 120 °C, desolvation gas (nitrogen) temperature at 400 °C and cone voltage 20 kV. For MS/MS experiments, nitrogen collision-induced dissociation was sat to both 10, 20 or 30 eV and run in separate runs. Data acquisition, pre-processing, visualization and reporting were performed in UNIFY 1.9.2 software (Waters Corporation, Manchester, UK) | | | | | | |

## Supplementary Table 3. Top 10 of metabolites correlated to adipic acid

| Pearson correlation | Proposed metabolite^a^ | RT^*^ | Mass | Data set | Adducts |
| --- | --- | --- | --- | --- | --- |
| 1.00 | Adipic acid^I^ | 2.29 | **145.0498** | Urine LC-MS (neg) | [M-H]- |
| 0.65 | Unknown^b^ | 3.16 | **318.1919**  319.196 | Urine LC-MS (pos) | [M-H]+  [M-2H]+ |
| 0.62 | Unknown | 3.42 | **245.1495** | Urine LC-MS (pos) | [M-H]+ |
| 0.62 | Unknown^b^ | 3.16 | 318.1919  **319.196** | Urine LC-MS (pos) | [M-H]+  [M-2H]+ |
| 0.61 | Unknown | 3.78 | 346.26 | Urine LC-MS (pos) | [M-H]+ |
| 0.56 | Unknown | 3.46 | 346.2236 | Urine LC-MS (pos) | [M-H]+ |
| 0.54 | Unknown | 3.56 | 259.1661 | Urine LC-MS (pos) | [M-H]+ |
| 0.53 | Unknown | 2.18-2.19 | - | Urine NMR | - |
| 0.53 | Unknown | 3.83 | 304.123 | Urine LC-MS (neg) | [M-H]- |
| 0.51 | Unknown | 4.03 | 245.139 | Urine LC-MS (neg) | [M-H]- |

^a^Identification level: I-V in accordance with the Metabolomics Standards Initiative classification scheme (1)

^b^Most likely same compound

^*^For NMR data the chemical shift (ppm) is reported.

## Supplementary Table 4 Top 10 of metabolites correlated to argininic acid

| Pearson correlation | Proposed metabolite^a^ | RT | Mass | Data set | Adducts |
| --- | --- | --- | --- | --- | --- |
| 1.00 | Argininic acid^I^ | 0.53 | **176.1041** | Urine LC-MS (pos) | [M-H]+ |
| 0.71 | α-keto-δ-guanidinovaleric acid^IV^ | 0.57 | **174.0891** | Urine LC-MS (pos) | [M-H]+ |
| 0.46 | 6-Guanidino-2-oxocaproic acid^IV^ | 0.61 | **188.1034** | Urine LC-MS (pos) | [M-H]+ |
| 0.44 | Unknown^b^ | 0.44 | **380.822**  238.8905  216.9093 | Urine LC-MS (neg) | [M-2H+Na]- 217.916  [M-H]- 217.916 |
| 0.43 | Unknown^b^ | 0.44 | 380.822  **238.8905**  216.9093 | Urine LC-MS (neg) | [M-2H+Na]- 217.916  [M-H]- 217.916 |
| 0.41 | Unknown | 1.02 | **133.0499** | Urine LC-MS (neg) | [M-H]- |
| 0.41 | Unknown | 1.98 | **588.2305** | Urine LC-MS (neg) | [M-H]- |
| 0.39 | Unknown^c^ | 0.44 | **322.8296**  180.8971 | Urine LC-MS (pos) | [2M+K]+ 141.933 / [4M+2K]2+ 141.933  [M+K]+ 141.933 |
| 0.38 | Unknown^c^ | 0.44 | **306.8559**  164.9232 | Urine LC-MS (pos) | [2M+Na]+ 141.933 / [4M+2Na]2+ 141.933  [M+Na]+ 141.933 |
| 0.38 | Unknown | 0.85 | **157.0134** | Urine LC-MS (neg) | [M-H]- |

^a^Identification level: I-V in accordance with the Metabolomics Standards Initiative classification scheme (1)

^b^Most likely same compound

^c^Most likely same compound

**Reference**

1. Sumner LW, Reily MD, Higashi R, Nicholls AW, Marriott P, Hardy N, et al. Proposed minimum reporting standards for chemical analysis. Metabolomics. 2007;3(3):211–21.
